# A rare case of synchronous multiple primary malignancies of breast cancer and diffuse large B-cell lymphoma that responded to multidisciplinary treatment: a case report

**DOI:** 10.1186/s40792-022-01456-z

**Published:** 2022-05-19

**Authors:** Yuichi Ueda, Yuko Makino, Taro Tochigi, Yoshikazu Ota, Hideki Hidaka, Takeshi Nakamura, Kiichiro Beppu, Jiro Ohuchida, Seiichi Odate, Soshi Terasaka, Takahiro Nishida, Masaki Yoshida, Ryuichiro Kimura, Kousuke Marutsuka, Naoki Otomo

**Affiliations:** 1 Department of Surgery, Miyazaki Prefectural Miyazaki Hospital, 5-30 Kitatakamatsu, Miyazaki, Miyazaki 880-8510 Japan; 2 Department of Internal Medicine, Miyazaki Prefectural Miyazaki Hospital, 5-30 Kitatakamatsu, Miyazaki, Miyazaki 880-8510 Japan; 3 Medical City Tobu Hospital, 3633-1 Tateno, Miyakonojo, Miyazaki 885-0035 Japan; 4 Department of Surgery, Faculty of Medicine, University of Miyazaki, 5200 Kihara, Kiyotake, Miyazaki, Miyazaki 889-1692 Japan; 5 Department of Diagnostic Pathology, Miyazaki Prefectural Miyazaki Hospital, 5-30 Kitatakamatsu, Miyazaki, Miyazaki 880-8510 Japan

**Keywords:** Multiple primary malignancies, Double cancer, Synchronous, Breast cancer, DLBCL

## Abstract

**Background:**

Multiple primary malignancies of breast cancer and diffuse large B-cell lymphoma (DLBCL) are rare. Here, we report a case of advanced breast cancer and DLBCL managed with multidisciplinary therapy preceded by surgery with a successful outcome.

**Case presentation:**

During a medical examination, a 71-year-old woman was diagnosed with a right breast mass, enlarged lymph nodes throughout the body, and a splenic tumor. The results of the clinical examination and imaging were suggestive of widely spread breast cancer with lymph node metastasis and malignant lymphoma with systemic metastasis. The histological evaluation of the biopsied breast tissue revealed human epidermal growth factor receptor 2 (HER2)-positive breast cancer, whereas the histological evaluation of the excised inguinal lymph node revealed DLBCL. ^18^F-FDG PET/computed tomography was performed, and it was determined that both breast cancer and DLBCL were in an advanced stage. Thus, mastectomy was performed, and the axillary lymph nodes showed mixed metastasis of breast cancer and DLBCL. Soon after, the R-CHOP therapy was initiated (375-mg/m^2^ rituximab, 2-mg/m^2^ vincristine, 50-mg/m^2^ doxorubicin, 750-mg/m^2^ cyclophosphamide, and 125-mg methylprednisolone). After irradiation of the spleen, trastuzumab was administered for 1 year.

**Conclusions:**

We experienced a case of combined breast cancer and DLBCL, which was difficult to treat because both were in advanced stages. Thorough staging of the malignancy and discussion by a multidisciplinary team are necessary to determine the optimal treatment strategy.

## Background

Multiple primary malignancies (MPMs) are present when two or more primary malignancies occur in a single patient. Although the incidence of MPMs has increased in recent years, cases of hematologic malignancies associated with solid tumors are relatively rare. In most cases, malignant lymphoma was diagnosed in the breast or ipsilateral axillary lymph node [1–4]. In this case of diffuse large B-cell lymphoma (DLBCL), the spleen was the primary tumor site. Breast cancer associated with abdominal malignant lymphoma is an extremely rare clinical entity. Here, we report a case of synchronous MPMs of advanced breast cancer and DLBCL that responded well to surgery followed by multidisciplinary treatment.

## Case presentation

A 71-year-old Japanese woman was diagnosed with a right breast mass during a specific medical checkup, after which she visited a local hospital. Contrast-enhanced computed tomography (CT) revealed a 5-cm mass in the right breast, enlarged lymph nodes throughout the body (bilateral axillary, right cervical, intra-abdominal, left iliac, and inguinal), and a splenic mass. The patient was referred to our hospital due to suspicion of advanced breast cancer. She had no family history of breast, ovarian cancers, or other malignancies.

**Fig. 1:**
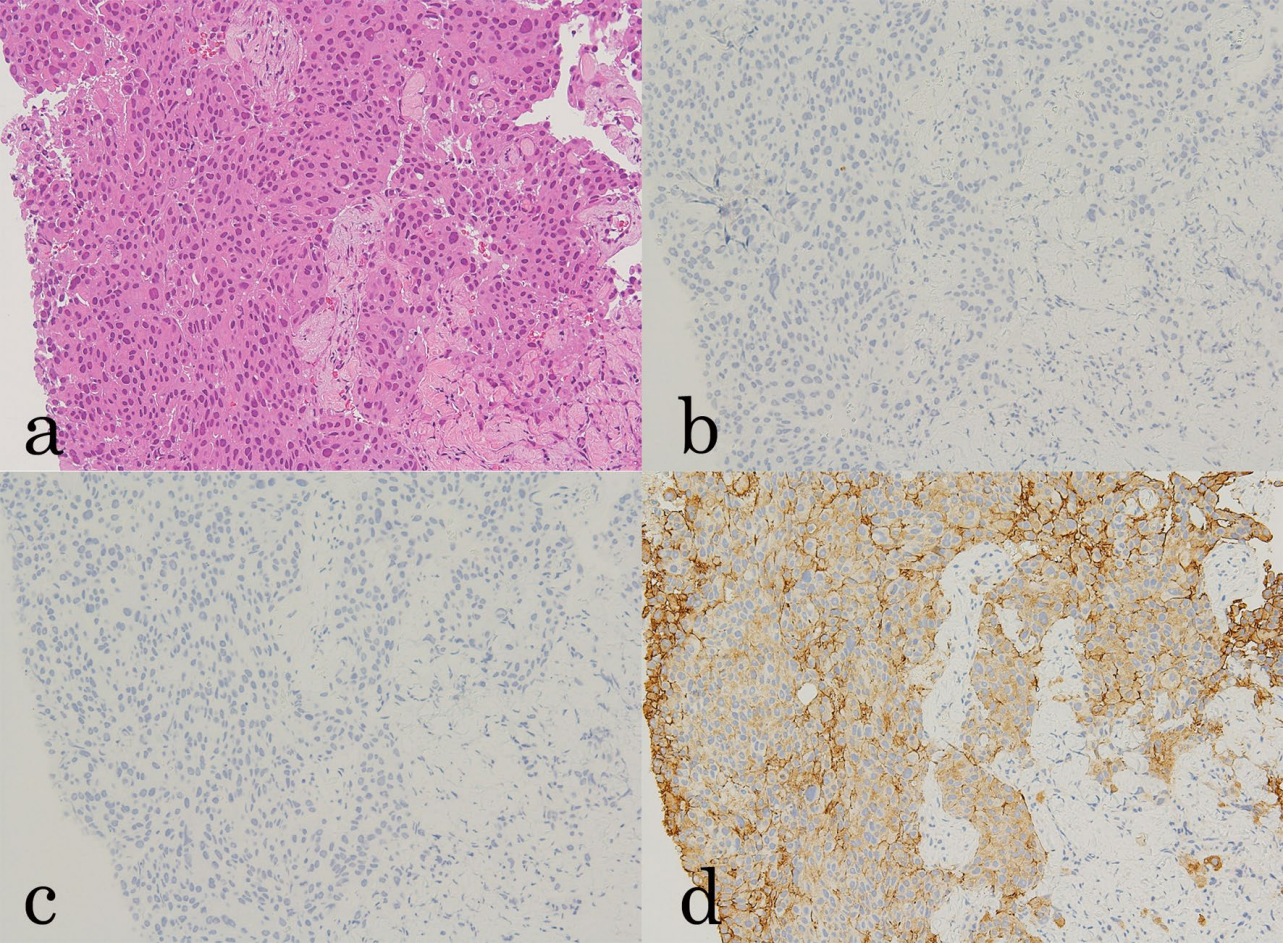
Histopathological examination of the breast tumor. **a** HE staining revealed invasive ductal carcinoma of the breast, histological grade III (×10 magnification). **b** ER negative (×10 magnification). **c** PgR negative (×10 magnification). **d** HER2 score3 (× 10 magnification)

Physical examination revealed erosions on the left nipple areola and a hard mass of approximately 5 cm just below the left nipple. Mammography revealed an indistinct mass just below the right nipple. Ultrasonography demonstrated a hypoechoic irregular mass, which was highly suspected to be malignant. Core needle biopsy revealed estrogen receptor-negative, progesterone receptor (PgR)-negative, and human epidermal growth factor receptor 2 (HER2)-positive invasive ductal carcinoma of the breast (Fig. 1).

**Fig. 2:**
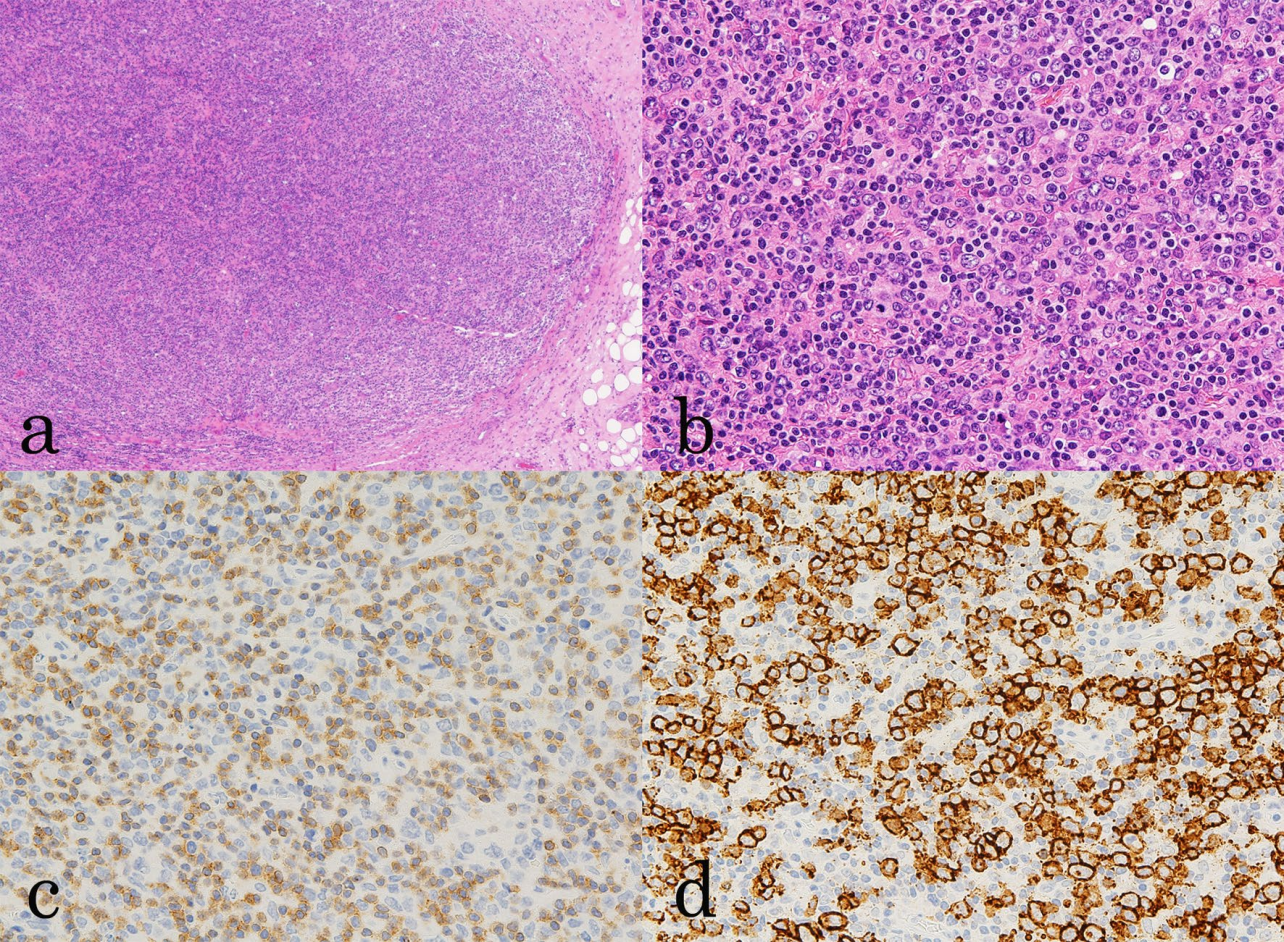
Histopathological examination of the inguinal lymph nodes. HE staining revealed the loss of the native lymph node architecture and diffused proliferation of large lymphocytes against a background of numerous small lymphocytes and histiocytes (**a** ×4 magnification; **b** ×20 magnification). Immunostaining for **c** CD3; **d** CD20. The large lymphoid cells are selectively positive for CD20, and numerous CD3-positive small T-lymphocytes are observed in background, supporting the diagnosis of diffuse large B-cell lymphoma (×20 magnification)

**Fig. 3:**
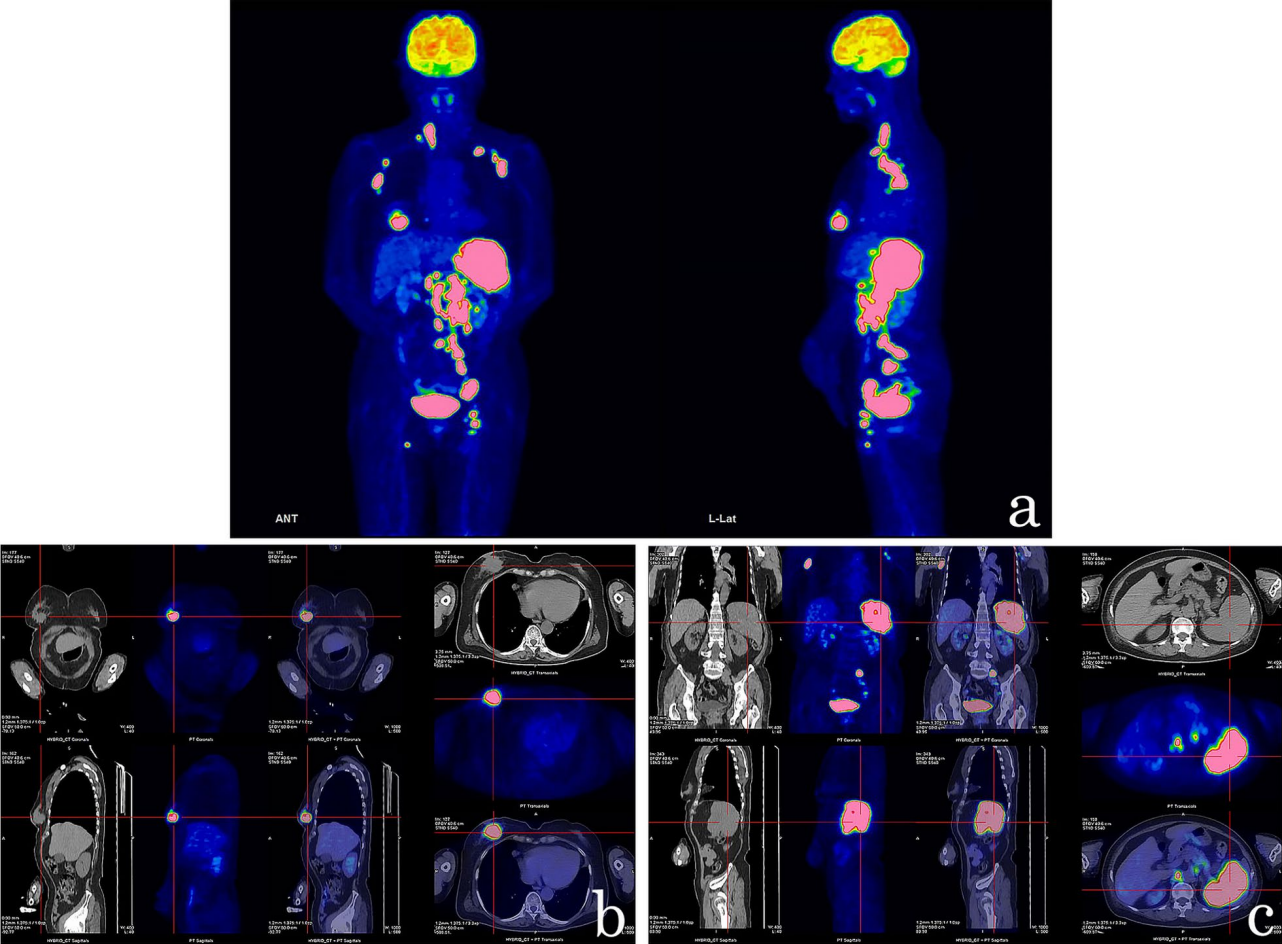
Pre-treatment PET/CT findings. PET demonstrated an accumulation of 18F fluorodeoxyglucose in the right breast, bilateral axillary, right cervical, intra-abdominal lymph nodes, spleen, and bilateral inguinal lymph nodes. The findings of the **a** whole body, **b** right breast, and **c** spleen are shown

Based on the enlarged lymph nodes throughout the body and the splenic mass confirmed by CT, we suspected malignant lymphoma and performed a biopsy to remove the inguinal lymph nodes. Histopathological analysis revealed that the patient had DLBCL (Fig. 2). ^18^F-FDG PET/CT was performed for staging and revealed an abnormal uptake in the right breast (SUVmax 13.3); the bilateral axillary; the right cervical, intra-abdominal, and bilateral inguinal lymph nodes; and the spleen (SUVmax 16.3) (Fig. 3).

Generally, patients are classified into high- and intermediate-risk groups according to the International Prognostic Index (IPI) and the Revised IPI (R-IPI) categories. Early treatment with the R-CHOP therapy was desirable. However, because the breast cancer was also advanced, mastectomy was performed as the initial treatment to predict prognosis and determine the treatment strategy. Multiple enlarged axillary lymph nodes were observed, but no enlarged supraclavicular lymph nodes were noted.

Therefore, radical surgery was highly likely, and axillary lymph node dissection was performed with total mastectomy.

**Fig. 4:**
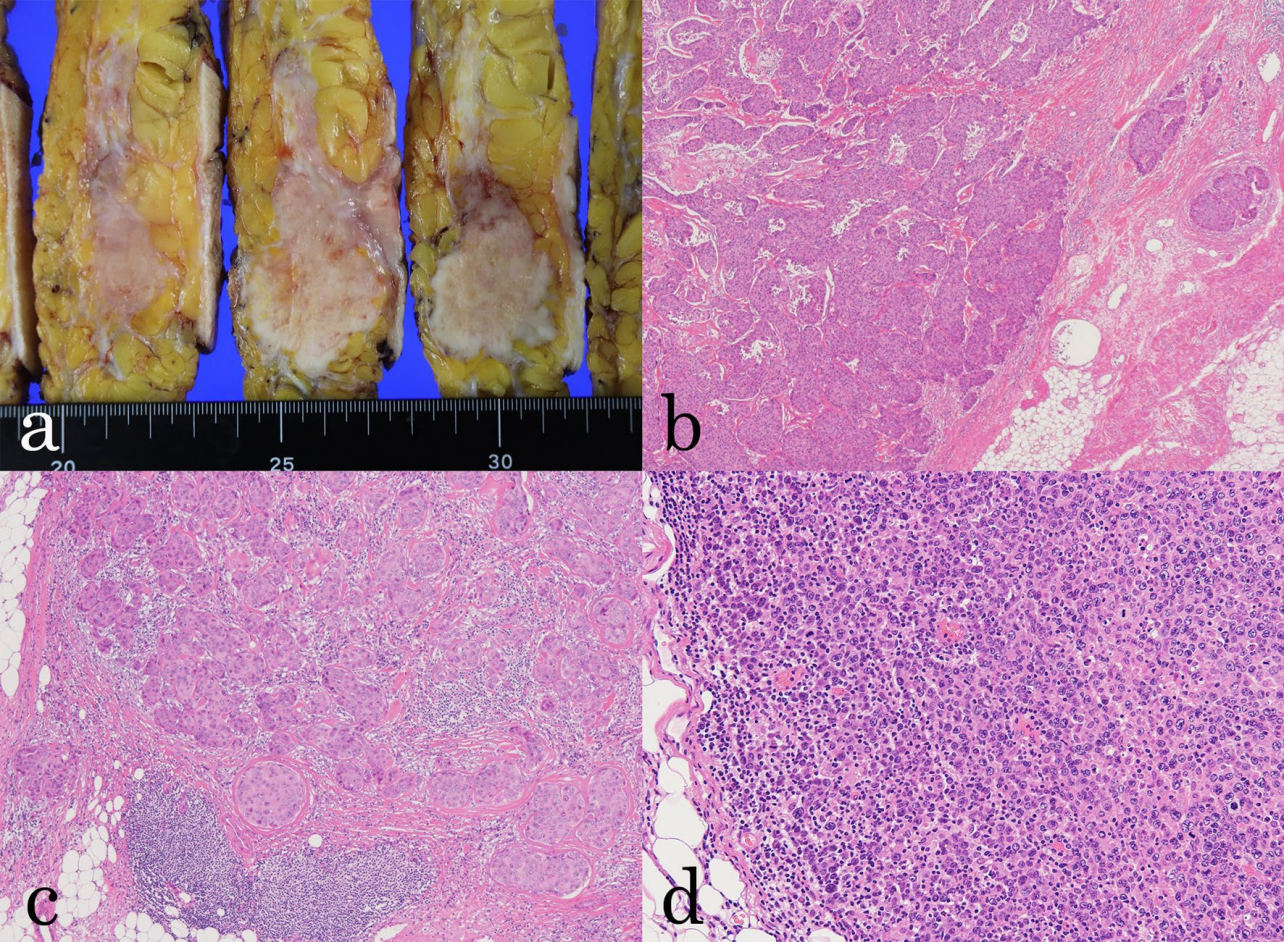
Macroscopic and microscopic findings of the resected specimen. **a** Surgical specimen of the right breast. **b** Pathological examination of a specimen from the breast showed invasive carcinoma (×4 magnification). **c** Pathological examination of the right axillary lymph node showed metastatic cells from the breast cancer (×10 magnification). **d** Pathological examination of the left axillary lymph node showed diffuse large B-cell lymphoma (×10 magnification)

The resected tissue showed histological findings similar to those of the biopsy specimen. The breast tumor was found to be an invasive carcinoma with a microscopic size of 68 × 33 mm and a histological grade of 3. Of the 14 removed axillary lymph nodes, 6 showed macrometastases of breast cancer, whereas 5 lymph nodes showed evidence of DLBCL. There were no mixed findings of breast cancer and DLBCL (collision carcinoma) in the same lymph node, and we determined that there was no possibility of distant lymph node metastasis of breast cancer (Fig. 4).

**Fig. 5:**
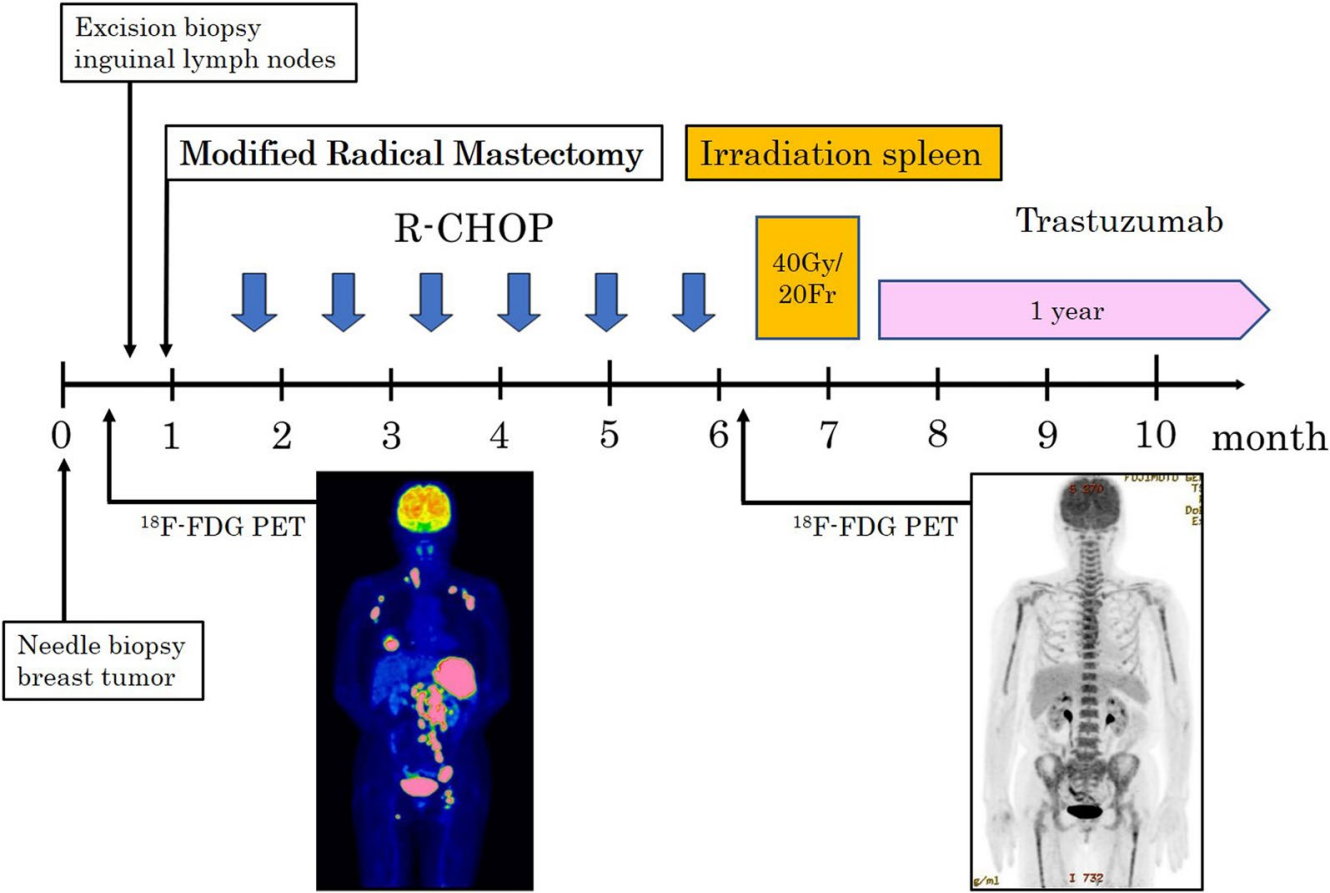
Treatment progress chart. After the completion of six courses of R-CHOP, PET–CT showed no lymphoma lesions or new lesions

Treatment was then initiated with the R-CHOP therapy (375-mg/m^2^ rituximab, 2-mg/m^2^ vincristine, 50-mg/m^2^ doxorubicin, 750-mg/m^2^ cyclophosphamide, and 125-mg methylprednisolone). After three courses of R-CHOP, simple CT revealed more than 50% tumor shrinkage by a sum of the products of diameter evaluation; after six courses of R-CHOP, no lymphoma lesions or new lesions were observed by PET–CT, and cardiovascular magnetic resonance evaluation was performed. After the completion of six courses of R-CHOP, no lymphoma lesions or new lesions were observed by PET–CT (Fig. 5). After the completion of chemotherapy, radiation (40 Gy/20 fractions) was applied to the spleen, which initially appeared as a bulky lesion. After irradiation, trastuzumab (Herceptin®) (8 mg/kg for the first time, 6 mg/kg for the second time) was administered for 1 year. We considered the addition of a taxane-based chemotherapeutic agent or the application of post mastectomy radiotherapy. However, these therapies were excluded due to the lack of patient agreement. The patient is being followed up with regular clinical examinations and has not exhibited any symptoms of recurrence more than 2 years after surgery.

## Discussion

MPMs are defined as the presence of two or more primary malignancies in a single patient, and specific criteria for the diagnosis of MPMs were provided by Warren and Gates in 1962 [5]. MPMs can be classified as synchronous or metachronous, depending on when they are diagnosed. Synchronous MPMs are diagnosed at the same time or within 2 months, whereas metachronous MPMs involve the discovery of a second tumor more than 6 months after the diagnosis of the first. Although the incidence of MPMs has increased in recent years, cases of hematologic malignancies associated with solid tumors are relatively rare. Gastric cancer has been reported to be the most common malignancy secondary to malignant lymphoma [6]. Reports of MPMs related to breast cancer and hematologic malignancies have also been recognized; moreover, breast cancer has been reported to be the most frequently diagnosed cancer after radiation therapy for Hodgkin lymphoma. On the contrary, the development of non-Hodgkin lymphoma (NHL) after breast cancer treatment is very rare [7].

In a retrospective review of synchronous MPMs (SPMs) in patients with breast cancer, Sas-Korczyńska et al. reported that of the 118,952 breast cancer patients treated between 1965 and 2014, SPMs were observed in only 112 (0.009%) patients. The most common type of synchronous primary malignancy was contralateral breast cancer (63.4%), and the most common synchronous malignancy other than breast cancer was of female genital origin (36.6%). The concurrence of lymphoid tissue tumors was only 3.6% [8]. This indicates that the simultaneous occurrence of breast cancer and malignant lymphoma is extremely rare.

In addition, malignant lymphoma was diagnosed in the breast or ipsilateral axillary lymph node in most cases [1–4]. In this case, the spleen was the primary site, and breast cancer associated with abdominal malignant lymphoma is an extremely rare clinical entity. We were able to find only two cases reported in the English literature [9, 10]; however, we were unable to find any published studies on successful treatment and a relatively long-term outcome [9, 10].

No consensus has been established with regard to prognostic factors for MPMs. One study reported that patients with SPMs have a shorter survival than those with metachronous MPMs [11]. Factors that contribute to the poor prognosis of patients with SPMs include the difficulty in accurately determining which malignancy is responsible for the lymphadenopathy. Furthermore, especially in patients with SPMs of breast cancer and NHL, the symptoms of B-cell NHL may be mistaken for menopausal symptoms. In addition, patients with operable SPMs can be treated with a single surgery and have less morbidity and better survival rate [12]. In this case, both the breast cancer and DLBCL were in an advanced stage, and it was difficult to determine the treatment plan. Surgery for breast cancer enabled accurate staging and contributed to optimal subsequent treatment. Deciding the timing of surgery for breast cancer is difficult as prior chemotherapy also requires DLBCL irradiation. The optimal treatment for patients with SPMs should be discussed by a multidisciplinary team and should consider the prognosis and curative potential of each.

## Conclusions

We present an extremely rare case of advanced stage DLBCL combined with breast cancer. Thorough staging of the malignancy and discussion by a multidisciplinary team are critical to determine the optimal treatment strategy.

## Data Availability

The data will not be shared, because there is no available data to be shared.

## Abbreviations

CT: Computed tomography
DLBCL: Diffuse large B-cell lymphoma
IPI: International Prognostic Index
MPM: Multiple primary malignancies
NHL: Non-Hodgkin lymphoma
SUV: Standardized uptake value
